# The complete mitochondrial genome of *Asymblepharus himalayanus* (Scincidae; Sauria) and its phylogenetic position within Scincidae

**DOI:** 10.1080/23802359.2021.1979430

**Published:** 2021-09-22

**Authors:** Shi-Yang Weng, Song Huang, Le Yang, Li-Fang Peng, Cong Wei, Jian-Chuan Li

**Affiliations:** a Tibet Plateau Institute of Biology, Lhasa, China; b Anhui Normal University, Anhui, China; c Tibet Museum of Natural Science, Lhasa, China

**Keywords:** Mitogenome, *Asymblepharus himalayanus*, Scincidae, Phylogeny

## Abstract

The complete mitochondrial genome of *Asymblepharus himalayanus*, has been determined for the first time by sanger sequencing. The overall length of the mitogenome is 17,304 bp and contains 13 protein-coding genes, 22 transfer RNA genes, 2 ribosomal RNA genes, and a putative control region. The total base composition is 31.2% for A, 27.0% for T, 14.4% for G, and 27.4% for C. The phylogenetic tree with the whole mitochondrial genome sequence of *A*. *himalayanus* together with 10 other related species belonging to the family Scincidae was reconstructed, in order to prove the validity of the mitogenome of *A*. *himalayanus.* Phylogenetic analysis indicated that *A. himalayanus* was not nested within *Scincella*, and further corroborated this species does not belong to the genus of *Scincella*.

*Asymblepharus himalayanus* (GÜNTHER, 1864), is one of the skinks in the Tibet Autonomous Region of China, which is distributed in the county of Purang (Che et al. 2020). First described by GÜNTHER in 1864, identified as *Eumeces himalayanus*. Due to the distribution area at the border and limited molecular genetic data, its classification status was always unclear and had been renamed to *Scincella himalayanus* (Cai et al. 2015; Wang et al. 2020), after combined molecular and morphological data, this species was designated as *A*. *himalayanus* (Che et al. 2020). Here, we determined the complete mitochondrial genome sequence of *A*. *himalayanus*.

The specimen of *A*. *himalayanus* was collected from Purang County, Ngari Prefecture, Xizang Autonomous Region, China (30.297 N, 81.187E, altitude 3925 m) on 30 June 2017. The voucher specimen was preserved and deposited in the Museum of Anhui Normal University (Voucher number: HSR18145; Contact person: LF Peng and email: lifang_peng@ahnu.edu.cn). Genomic DNA was extracted from macerated liver or muscle tissues using an Ezup Column Animal Genomic DNA Purification Kit (Map Biotech, China), according to the protocols of the manufacturer.

The mitogenome sequence was amplified by polymerase chain reaction (PCR). The PCR products were sequenced using Sanger sequencing by a commercial company (Map Biotech, China). Sequences were assembled manually using SeqMan in Lasergene v15.1 (DNASTAR, Inc., Madison, Wisconsin, USA). The positions of RNA genes and protein-coding genes were identified by MITOS, Candidate protein-coding genes were found by detecting congruences in the results of blastx searches against the amino acid sequences of the annotated proteins of other related mitochondrial genomes found in the NCBI RefSeq. A postprocessing step detects the start and stop codons, duplicates, and hits belonging to the same transcript (Bernt et al. 2013).

The total length of the complete mitogenome (Genbank accession number: MN885892) of *A*. *himalayanus* was sequenced to be 17,304 bp which consisted of 13 typical vertebrate protein-coding genes (PCGs), 22 transfer RNA (tRNA) genes, 2 ribosomal RNA (rRNA) genes and one control region. The base composition was 31.2% for A, 27.0% for T, 14.4% for G, and 27.4% for C. complete mitogenome was encoded on the H-strand except for the ND6 gene and eight tRNA genes, which were encoded on the L-strand. The 22 tRNA genes ranged in size from 66 to 75 bp. Among the mitochondrial protein-coding genes, the ATP8 was the shortest, while the ND5 was the longest. The 12 s rRNA (949 bp) and 16 s rRNA (1495 bp), were located between the tRNA-Phe and tRNA-Leu gene and separated by the tRNA-Val gene. The gene order, contents and base composition were identical to those found in typical vertebrates (Boore 1999; Sorenson et al. 1999).

In order to help clarifying its phylogenetic position, we used the whole mitochondrial genome sequences of *A*. *himalayanus* and other 10 related species to perform a phylogenetic analysis. These species were as follows: *Scincella reevesii, S*. *huanrenensis*, *S*. *vandenburghi*, *S*. *modesta*, *Tropidophorus hangman*, *Scincus scincus*, *Plestiodon egregious*, *Sphenomorphus indicus*, *Isopachys gyldenstolpei*, *Ateuchosaurus chinensis* (outgroup). We aligned these sequences using MAFFT 7.307 (Katoh et al. 2019). Maximum likelihood (ML) methods was used to reconstruct phylogenetic tree (Figure 1) in http://www.phylo.org/portal2/login!input.action. Phylogenetic analysis indicated that *A. himalayanus* was sister to the groups of *Tropidophorus hangman*, *Isopachys gyldenstolpei*, and *Scincella spp*., not nested within *Scincella*. We further corroborated this species does not belong to the genus *Scincella*. Given the limited genetic data used, our results did not provide enough resolution regarding phylogenetic relationships between the genera *Scincella* and *Asymblepharus*. The species division of intra-genera and the relationships with other genera are still unclear, more specimens and molecular data are needed in the future.

**Figure 1.**
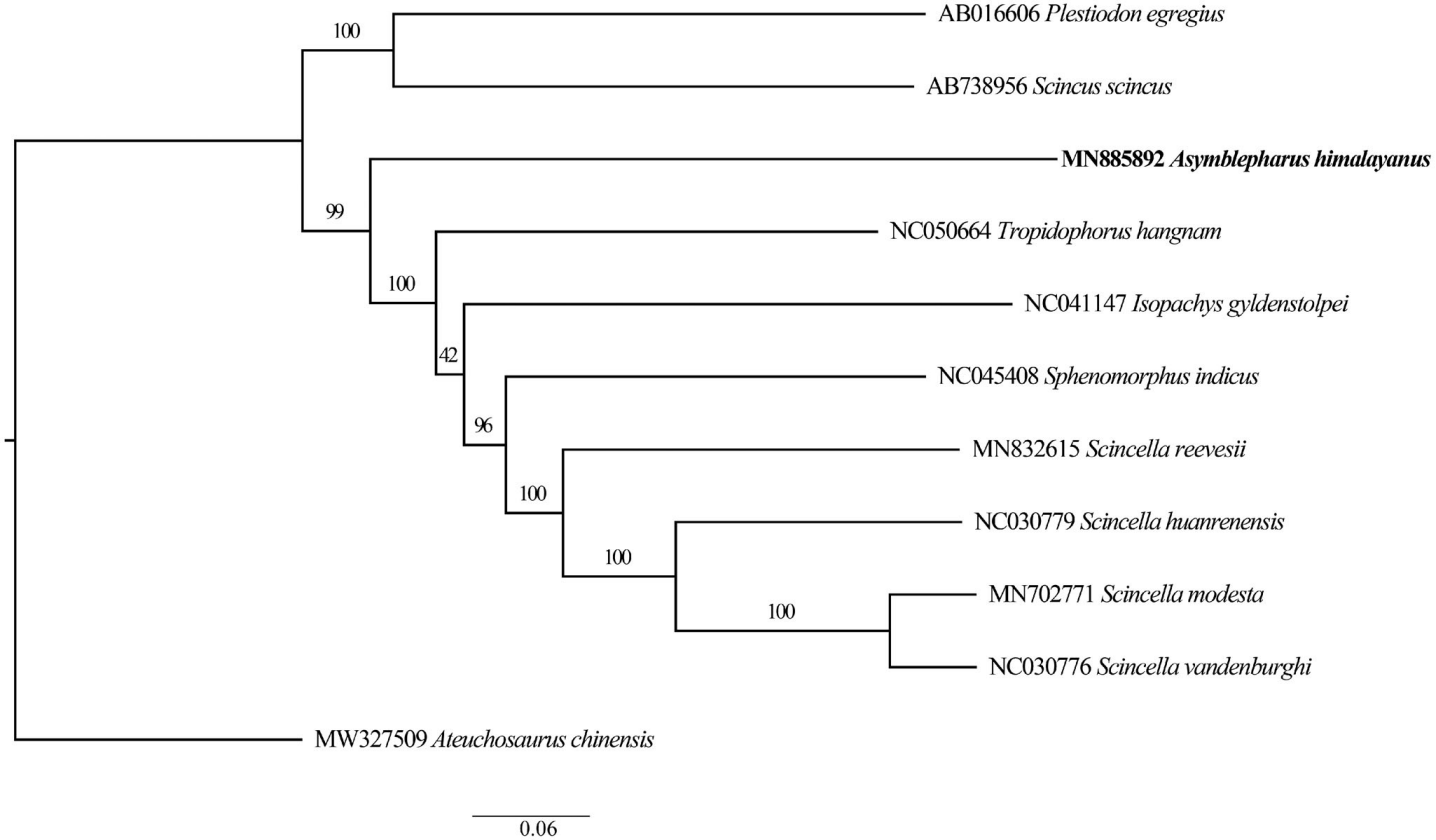
A maximum likelihood (ML) tree of *A*. *himalayanus* in this study and other 10 related species was reconstructed based on the dataset of the whole mitochondrial genome. Values above branches correspond to ML bootstrap percentages.

## Data Availability

The complete mitochondrial genome sequence and annotation of *A*. *himalayanus* that support the findings of this study are available in GenBank of NCBI at [https://www.ncbi.nlm.nih.gov] under the accession NO. MN885892.
